# Supplementary material for: Adolescent sexual and reproductive health literacy in Saudi Arabia: The roles of school type and maternal education
Source: PLoS One. 2026 May 21;21(5):e0347429. doi: 10.1371/journal.pone.0347429 (PMC13193505; doi:10.1371/journal.pone.0347429)
Supplement: Attachment — Submitted filename: Response to Reviewers.pdf [file pone.0347429.s002.pdf]

We sincerely thank the reviewer for the thoughtful and constructive feedback. We appreciate the recognition of the importance of this topic in the Saudi context. We have carefully revised the manuscript to address all conceptual and methodological concerns. Below, we respond point-by-point.

Reviewer Comment:

Clarify why only ≥18-year-old participants were included and justify the exclusive focus on female students.

Response:

We have now clarified this in both the **Introduction** and **Section 2.2 (Study Population).** The inclusion of students aged 18 years and above was primarily due to ethical and administrative requirements. In Saudi Arabia, research involving minors requires additional institutional and parental approvals that can significantly limit feasibility in sensitive topics such as SRH. Focusing on the oldest segment of adolescence allowed independent informed consent while still aligning with the WHO definition of adolescence (10–19 years).

The decision to include only female students was culturally and contextually grounded. In Saudi Arabia, reproductive health communication is strongly gender-segregated, and mothers are typically the primary communicators with daughters regarding SRH matters. Given this socio-cultural structure and the focus on mother–daughter communication, a female-only sample allowed for culturally coherent and context-specific analysis. We have now explicitly justified this approach and acknowledged the exclusion of male adolescents as a limitation.

Reviewer Comment:

Provide detailed description of the questionnaire and report psychometric properties.

Response:

We have expanded Section 2.3 to provide:

Clear description of domains and item structure Scoring procedure and possible score ranges Explanation of composite knowledge score construction

Additionally, internal consistency reliability (Cronbach's alpha) for each domain has now been calculated and reported in the revised manuscript. The full Arabic questionnaire has been added as a supplementary file to ensure transparency and reproducibility.

Reviewer Comment:

Clarify inconsistency between logistic regression and OLS models; address clustering and assumptions.

Response:

We thank the reviewer for identifying this inconsistency. The final analytical model used was multivariable OLS regression with HC3 robust standard errors. Logistic regression was mentioned in an earlier draft and has now been removed for consistency.

we Clarified model selection rationale Explained why OLS was appropriate for continuous composite knowledge scores Noted that clustering at the school level was not explicitly modeled and acknowledged this as a limitation Added a brief statement describing evaluation of parametric assumptions These revisions improve methodological clarity.

Reviewer Comment:

Strengthen alignment with international SRH frameworks and expand beyond biomedical framing.

Response:

We have substantially revised the Introduction to integrate internationally recognized frameworks, including: WHO definitions of sexual and reproductive health Rights-based perspectives grounded in global standards Comprehensive sexuality education frameworks

The manuscript now adopts a multidimensional understanding of SRH that includes biological, emotional, relational, social, and rights-based components, rather than focusing solely on risk prevention. This strengthens theoretical grounding and situates the study within global best practices.

Reviewer Comment:

Clarify selection criteria and methodological basis for comparative synthesis.

Response:

We have clarified that Table 6 represents a structured narrative comparative synthesis rather than a systematic review. We now describe:

Criteria for selecting studies (peer-reviewed, 2005–2025, focus on female adolescents in conservative/MENA contexts) Approach to extracting comparable domains Limitations related to heterogeneity in study designs

We also explicitly acknowledge that the table includes heterogeneous methodologies and caution interpretation accordingly. We have expanded the Limitations section to include: Social desirability bias due to sensitive topic Restricted age range (≥18 only) Exclusion of male

adolescents Lack of full psychometric validation within the present sample Absence of clustering adjustment

This strengthens transparency and scientific rigor. The Data Availability Statement has been revised. The anonymized dataset and full questionnaire have now been prepared for submission as supplementary files, in compliance with PLOS ONE’s data-sharing requirements.
